# Retrospective Analysis of Vesicourethral‐Anastomosis Stricture/Urethral Stricture After Robotic‐Assisted Laparoscopic Radical Prostatectomy With and Without Radiotherapy

**DOI:** 10.1111/iju.70339

**Published:** 2026-01-11

**Authors:** Kinan Massouh, Katharina Leucht, Lutz Leistritz, Marc‐Oliver Grimm

**Affiliations:** ^1^ Department of Urology Jena University Hospital, Friedrich‐Schiller University Jena Germany; ^2^ Comprehensive Cancer Center Central Germany (CCCG) Munich Germany; ^3^ Institute of Medical Statistics, Computer and Data Sciences Jena University Hospital Jena Germany

**Keywords:** prostate cancer, radiotherapy, robotic‐assisted radical prostatectomy, urethral stricture, vesicourethral anastomosis stricture

## Abstract

**Background and Objective:**

Vesicourethral anastomotic stenosis and/or urethral stenosis (VUAS/US) is a complication of robotic‐assisted radical prostatectomy (RARP) for prostate cancer. We aimed to evaluate the incidence of VUAS/US after RARP and to identify potential risk factors.

**Materials and Methods:**

We performed a retrospective assessment of clinical records of patients with RARP as primary prostate cancer treatment (January 2011–December 2018) and investigated associations between VUAS/US formation and radiotherapy, pT‐stage, tumor margins, Gleason score, nerve‐sparing, and postoperative duration of bladder catheterization. Statistical analysis was performed via uni‐ and multivariable cox regression; risk estimation was done with the Kaplan–Meier method and log‐rank test.

**Results and Limitations:**

809 patients were included in the study. Median clinical follow‐up was 61.0 months (IQR 50.0–75.0) and 175 (22%) patients received radiotherapy. VUAS and US were recorded in 19 (2.3%) and 10 (1.2%) patients, respectively. Whereas in univariable analysis radiotherapy, pT‐stage ≥ pT3a, higher Gleason score, positive tumor margins, nerve‐sparing, and prolonged duration of bladder catheterization were significant risk factors, in a multivariable analysis only radiotherapy (*p* = 0.003) and prolonged duration of bladder catheterization (*p* = 0.0309) proved to be independently predictive. Estimated 5‐year risk of VUAS/US formation was lower without than with radiotherapy (2.1% [95% CI: 0.9–3.3] vs. 7.3% [95% CI: 3.4–11]) and with normal compared to prolonged bladder catheterization (2.6% [95% CI: 1.4–3.8] vs. 9.9% [95% CI: 2.3–18]). Retrospectivity and the limited number of events were our major limitations.

**Conclusions and Clinical Implications:**

Low incidence of VUAS/US in RARP. Patients undergoing radiotherapy or requiring prolonged catheterization should be explicitly informed about the risk of VUAS/US and about symptoms. Also, physicians must be aware.

## 1 Introduction

Prostate cancer (PCa) represents the most frequent, solid malignant tumor among men in the Western hemisphere [1]. The most common treatment option in localized disease is radical prostatectomy (RP) with, however, numerous known side effects including urinary incontinence and erectile dysfunction. After removal of the prostate, the bladder is anastomosed to the membranous urethra, which may cause another, albeit less frequently occurring side effect: vesicourethral anastomosis stricture (VUAS) [2, 3]. The incidence of urethral strictures (US) and stenoses requiring treatment after PCa therapy (surgery and radiotherapy) is 5.2%, based on the CaPSURE database [4], but has also been reported to be up to 15% in patients with open surgery [5, 6]. If performing robotic‐assisted prostatectomy (RARP), however, incidences are lower [7]. In a Swedish prospective trial comparing retropubic RP and RARP, symptomatic stenosis developed in 3.6% (34/942) and 1.3% (37/2764) of evaluable men within 24 months post procedure [8].

Although exact etiology of development of stenosis remains unknown, several risk factors have been described. These include obesity, smoking, diabetes, and hypertension [9], all of which may lead to a reduced microvasculature and thus to prolonged healing of vesicourethral anastomosis (VUA). Further risk factors for VUAS represent transurethral resection of the prostate prior to RP, large prostatic volume, and intraoperative factors like blood loss, mismatch, and tension on the anastomosis [9, 10, 11]. On the other hand, running VUA sutures and a robot‐assisted surgical approach are supposed to lower the risk [11, 12]. Most VUAS occur within the first 6 months after surgery, with a decreasing incidence thereafter and a negligible risk from 2 years post‐RP [4].

Few data exist on the association between radiotherapy and VUAS/US formation after RP. In general, RP featured an increased risk for stricture formation vs. radiotherapy within the first 24 months post‐procedure. However, with longer follow‐up radiotherapy had higher stricture rates than surgery [13]. Radiotherapy being applied in the adjuvant or salvage setting after RP was reported to cause strictures in approximately 3% of patients [14]. This rate appears to be reducible by delaying adjuvant radiotherapy to > 9 months [15]. However, in a retrospective analysis that assessed clinical records of patients with RP, postoperative radiotherapy did not significantly increase the incidence of VUAS (4/25 [16%] patients with vs. 36/233 [15%] without radiotherapy, *p* = 0.599) [16].

The primary objective of our study was to investigate the occurrence of VUAS/US in a large cohort of patients who underwent RARP at our hospital, and to determine the impact of radiotherapy and further risk factors.

## 2 Patients and Methods

We retrospectively reviewed the medical records of consecutive patients that underwent RARP for PCa at Jena University Hospital (Jena, Germany) between January 2011 and December 2018, with a minimum clinical follow‐up of 12 months. Patients were contacted by mail and asked whether postoperative complications had occurred and whether additional surgical interventions involving the bladder or urethra were required following RARP. Additional information was requested from the treating urologists when necessary. Such requests were made only after the treating physician had been formally released from the duty of confidentiality in writing.

### 2.1 Surgical Procedures

RARP was performed using a daVinci Si Surgical System (Intuitive Surgical Inc., CA, USA) with a transperitoneal approach. Nerve‐sparing was performed based on clinical staging. A running VUA was made using 15 cm 3–0 monofilament sutures previously tied together to create a double‐armed suture. The needles used to complete the anastomosis were passed through the urethral and bladder neck about 8–12 times followed by tying the suture ends together at the 12 o'clock position. The bladder was irrigated through the final Foley catheter to confirm a watertight anastomosis.

Cystography was performed 5–8 days post‐surgery, and when VUA healing was confirmed, the Foley catheter was removed.

VUAS and US were diagnosed in most cases based on clinical suspicion and was confirmed endoscopically. Patients reporting hesitancy, slow stream, frequency, sensation of incomplete emptying or urinary retention underwent urine analysis, flow rate profile, postvoid residual ultrasound testing and cystoscopy depending on the initial evaluation. VUAS and US was diagnosed by direct vision with flexible cystoscopy and the inability to pass the cystoscope into the bladder through a lumen of < 16 F.

In case of radiotherapy, the surgical site was irradiated in five fractions per week with a dose of 1.7–2.0 Gy per fraction.

### 2.2 Assessed Parameters

We assessed the time‐dependent formation of VUAS/US in our population. After an extensive literature review, we have identified the potential risk factors radiotherapy (no radiotherapy vs. radiotherapy), T‐staging (≤ pT2c vs. ≥ pT3a), Gleason score (GS) (≤ 7a vs. ≥ 7b), tumor margins (R0 vs. R1), nerve‐sparing (none vs. unilateral vs. bilateral), and postoperative catheter length of stay (normal [≤ 8 days] vs. prolonged [> 8 days]). These risk factors were available from the clinical records and analyzed as such as well as in association with the presence or absence of radiotherapy.

### 2.3 Statistical Analysis

Statistical analysis was performed using SPSS Statistics version 29 (IBM Corp., NY, USA) and R version 4.5.1 (The R Foundation for Statistical Computing, Vienna, Austria). Categorical variables were described by number and percentage, continuous variables by medians and interquartile ranges (IQRs). Time‐to‐VUAS/US per group were assessed by the Kaplan–Meier method and compared using the log‐rank‐test or, for parameters with multiple ordered groups, the trend log‐rank‐test [17, 18]. Multivariable Cox regression with Firth's penalized likelihood correction was performed to identify independent prognostic factors (function coxphf implemented in the R package coxphf). Firth's correction was used due to the low number of events observed (*n* = 29) being insufficient to support a multivariable Cox regression model with six covariates. As sensitivity analyses the risk factor of catheter lengths of stay was assessed as a continuous covariate in a Cox proportional hazards model. Results were considered statistically significant at *p* < 0.05.

## 3 Results

### 3.1 Patient Characteristics

In this study, 809 consecutive patients undergoing RARP at Jena University Hospital were included. The main patient characteristics are summarized in Table 1. The median age at the time of surgery was 66 years (IQR 60–70). A total of 175 (22%) patients received radiotherapy. The median interval between surgery and radiotherapy was 9.4 months (IQR 2.0–10), and the median radiation dose was 70 Gy (IQR 70–70). Of all patients, 541 (67%) had ≤ pT2c, whereas 268 (33%) had ≥ pT3a. Regarding GS, 486 (60%) patients had GS ≤ 7a, and 323 (40%) GS ≥ 7b. Negative surgical margins were observed in 597 (74%) patients. A total of 149 (18%) patients underwent surgery without nerve‐sparing, 138 (17%) had unilateral, and 522 (65%) bilateral nerve‐sparing. The median postoperative catheterization time was 6 days (IQR 5–7), with 745 (92%) patients having a normal catheter stay of ≤ 8 days. At Jena University Hospital, cystography is routinely performed between postoperative days five and eight and serves as the basis for determining the timing of catheter removal. Removal within this timeframe is considered standard practice (i.e., a normal catheter duration of ≤ 8 days). When the cystogram shows extravasation or other abnormalities, prolonged catheterization (> 8 days) is required. This represents institutional practice.

**TABLE 1:** Patient characteristics.

Characteristic	*N* = 809
Median age, years (IQR)	66 (60–70)
No radiotherapy, *n* (%)	634 (78)
Radiotherapy, *n* (%)	175 (22)
Adjuvant, *n* (% of total radiotherapy)	135 (77)
Salvage, *n* (% of total radiotherapy)	39 (22)
Primary, *n* (% of total radiotherapy)	1 (0.6)
Median time between RARP and radiotherapy, months (IQR)	9.4 (2.0–10)
Median radiotherapy dose administered, Gy (IQR)	70 (70–70)
pT‐stage	
≤ pT2c, *n* (%)	541 (67)
≥ pT3a, *n* (%)	268 (33)
Gleason score	
≤ 7a, *n* (%)	486 (60)
≥ 7b, *n* (%)	323 (40)
Tumor margins	
R0, *n* (%)	597 (74)
R1 and Rx, *n* (%)	212 (26)
Nerve‐sparing	
None, *n* (%)	149 (18)
Unilateral, *n* (%)	138 (17)
Bilateral, *n* (%)	522 (65)
Median post‐operative catheter length of stay, days (IQR)	6 (5–7)
Normal, *n* (%)	745 (92)
Prolonged, *n* (%)	64 (7.9)

Abbreviations: IQR, interquartile range; R0, negative tumor margin; R1, positive tumor margin; RARP, robot‐assisted radical prostatectomy.

### 3.2 Univariable Analyses

**FIGURE 1:**
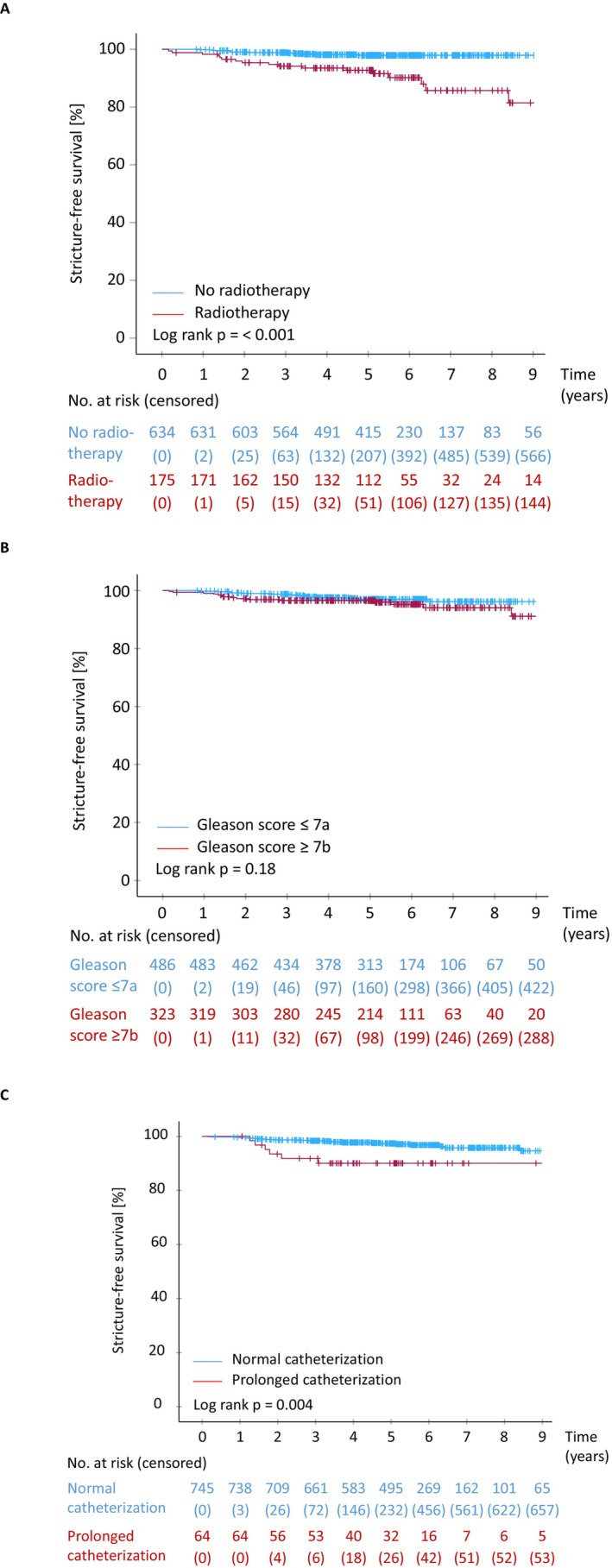
Kaplan Meier analysis of stricture‐free survival (time between robotic‐assisted radical prostatectomy and formation of vesicourethral anastomosis strictures [VUAS] and/or urethral strictures [US]). (A) No radiotherapy vs. radiotherapy; (B) Gleason score ≤ 7a versus ≥ 7b, (C) Duration of catheterization normal (≤ 8 days) versus prolonged (> 8 days).

The median duration of clinical follow‐up was 61.0 months (IQR 60.0–75.0). Overall, 19 (2.3%) patients developed VUAS and 10 (1.2%) US during the observation period. VUAS/US occurred in 12/634 (1.9%) patients without radiotherapy and in 17/175 (9.7%) patients with radiotherapy. The estimated 5‐year risk of developing VUAS/US was 2.1% (95% CI: 0.9–3.3) with and 7.3% (95% CI: 3.4–11) without radiotherapy (*p* < 0.001, Figure 1A, Table 2).

**TABLE 2:** Univariable analysis of time to formation of vesicourethral anastomosis strictures (VUAS) and/or urethral strictures (US).

Variable		No. of patients	VUAS/US, %^a^	95% CI	*p*
Radiotherapy	No	634	2.1	0.9–3.3	< 0.001
	Yes	175	7.3	3.4–11	
pT‐stage	≤ pT2c	541	2.2	0.8–3.6	0.002
	≥ pT3a	268	5.2	2.5–7.9	
Gleason score	≤ 7a	486	2.9	1.4–4.6	0.18
	≥ 7b	323	3.5	1.5–5.5	
R‐status	R0	597	2.4	1.0–3.8	0.006
	R1	212	5.5	2.4–8.6	
Nerve‐sparing	None	149	4.5	1.0–8.0	0.009
	Unilateral	138	5.1	1.2–9.0	
	Bilateral	522	2.4	1.0–3.8	
Duration of bladder catheterization	Normal	745	2.6	1.4–3.8	0.004
	Prolonged	64	9.9	2.3–18	

Abbreviations: CI, confidence interval; R0, negative tumor margin; R1, positive tumor margin.

^a^ Derived via Kaplan–Meier method, after 5 years.

A higher pathological T stage was associated with an increased estimated 5‐year risk of VUAS/US in univariable analysis (≤ pT2c: 2.2% [95% CI: 0.8–3.6] vs. ≥ pT3a: 5.2% [95% CI: 2.5–7.9], *p* = 0.002). Similarly, a higher GS increased the estimated 5‐year risk: in patients with GS ≤ 7a, the estimated 5‐year risk was 2.9% (95% CI: 1.4–4.6), compared with 3.5% (95% CI: 1.5–5.5) in patients with GS ≥ 7b (*p* = 0.18; Figure 1B, Table 2).

Patients with positive surgical margins (R1) also showed higher rates of VUAS/US compared with those with negative margins (R0), with estimated 5‐year risks of 5.5% (95% CI: 2.4–8.6) and 2.4% (95% CI: 1.0–3.8, *p* = 0.006), respectively. Nerve‐sparing had a significant impact as well: the estimated 5‐year risks were 4.5% (95% CI: 1.0–8.0) without nerve‐sparing, 5.1% (95% CI: 1.2–9.0) with unilateral, and 2.4% (95% CI: 1.0–3.8) with bilateral nerve‐sparing (*p* = 0.009). Kaplan–Meier curves began to diverge after the 5‐year mark, indicating the lowest long‐term risk with bilateral nerve‐sparing, followed by unilateral and no nerve‐sparing (data not shown). Prolonged duration of bladder catheterization was also associated with an increased estimated 5‐year risk of VUAS/US formation (normal: 2.6% [95% CI: 1.4–3.8] vs. prolonged: 9.9% [95% CI: 2.3–17.5], *p* = 0.004; Figure 1C). In a univariable Cox regression analysis, catheterization duration (as a continuous covariate) showed a significant association with time‐dependent VUAS/US formation (*p* < 0.001).

### 3.3 Multivariable Analyses

After univariable screening of all candidate variables, a multivariable analysis was performed. Despite its lack of statistical significance in the univariable analysis, the GS represents a clinically relevant potential confounder. Using a 5% significance level for univariable screening risks excluding relevant predictors. A 25% threshold has been shown to be appropriate [19] and was applied in our analyses. Therefore, it is also reasonable from a statistical perspective not to exclude the GS.

Despite the large sample size, the number of events was relatively small. Therefore, the multivariable analysis was performed using Firth's correction to the score function of the Cox model. In the multivariable model, only radiotherapy and prolonged (vs. normal) catheterization were independently associated with a higher risk (radiotherapy: hazard ratio [HR] 4.00 [95% CI: 1.60–10.21], *p* = 0.003; prolonged catheterization: HR 2.97 [95% CI: 1.13–6.83], *p* = 0.030, respectively; Table 3). pT‐stage, R‐status, GS, and application of nerve‐sparing (both for no NS vs. unilateral and for no NS vs. bilateral) were not associated with the time‐to‐VUAS/US.

**TABLE 3:** Multivariable analysis of time to formation of vesicourethral anastomosis strictures (VUAS) and/or urethral strictures (US).

Variable	Hazard ratio	95% CI	*p*
Radiotherapy (vs. no radiotherapy)	4.00	1.60–10.21	0.003
pT‐stage (≥ pT3a vs. ≤ pT2c)	1.48	0.57–3.82	0.42
Gleason score (≥ 7b vs. ≤ 7a)	0.76	0.33–1.78	0.53
R‐status (R0 vs. R1)	1.17	0.50–2.78	0.72
Nerve‐sparing (unilateral vs. none)	1.14	0.39–3.15	0.80
Nerve‐sparing (bilateral vs. none)	0.65	0.26–1.63	0.35
Duration of bladder catheterization (prolonged vs. normal)	2.97	1.13–6.83	0.030

Abbreviations: CI, confidence interval; R0, negative tumor margin; R1, positive tumor margin.

### 3.4 Subgroup Analyses

When restricting the analysis to patients without radiotherapy, univariable analysis demonstrated a significant association between time‐to‐VUAS/US and duration of bladder catheterization. Patients with a normal catheterization duration exhibited a lower estimated 5‐year risk of VUAS/US compared with those with prolonged catheterization duration (1.5% [95% CI: 0.5–2.5] vs. 8.9% [95% CI: 0.7–17], *p* < 0.001; Table S1). In contrast, other potential risk factors (pT‐stage, GS, R‐status and nerve‐sparing technique) did not show a significant association with VUAS/US development within the cohort without radiotherapy (*p* = 0.12, *p* = 0.64, *p* = 0.97, *p* = 0.56, respectively). Among patients receiving radiotherapy, none of the assessed variables demonstrated a significant association with the time‐to‐VUAS/US.

We furthermore assessed the impact of radiotherapy on time‐to‐VUAS/US within the predefined subgroups according to each risk factor. Radiotherapy significantly increased the estimated 5‐year risk of VUAS/US in patients with both ≤ pT2c and ≥ pT3a, GS ≤ 7a and GS ≥ 7b, R0 and R1 resection margins, no or bilateral nerve‐sparing, and normal duration of bladder catheterization (*p* < 0.05 each, see Table S1).

## 4 Discussion

Our retrospective analysis showed that patients who underwent RARP for PCa had an increased risk of VUAS/US if they additionally underwent radiotherapy or had a prolonged catheter stay.

In our study, the median follow‐up period is very long, about 5.1 years, which is a very positive aspect compared to other studies. Most other studies reported median follow‐up times ranging from 1 to 4.5 years [4, 5, 6, 8, 9, 11, 12, 16, 17]. There is one study that is similar to ours, with a median follow‐up of approximately 4.2 years, but it included fewer patients (*n* = 258) [16] compared to our larger sample size.

Numerous studies have shown that a laparoscopic approach is advantageous with regard to the formation of VUAS as a complication of RP. A retrospective analysis found, for example, that laparoscopy (*n* = 1134, including 97 robotic‐assisted cases) significantly reduced symptomatic anastomotic strictures compared to open surgery (*n* = 3458) [20]. Similar findings were reported for RARP versus an open approach in the prospective LAPPRO study (2.2‐fold increased risk for the open approach) [8] as well as in a large retrospective analysis (odds ratio [OR] 0.39; *p* < 0.01) [21]. In the overall population of this latter retrospective analysis with different surgical techniques applied, adjuvant radiotherapy was one factor associated with increased VUAS formation in a multivariable analysis (OR 1.66, *p* = 0.002) [21]. Our own findings emphasize the negative impact of adjuvant radiotherapy in an exclusive RARP population, since the estimated 5‐year risk for VUAS/US was 4.0‐times higher in patients with as compared to those without radiotherapy. In the LAPPRO trial clearly fewer patients than in our study received radiotherapy (3.8% during 0–12 months and 4.3% during 12–24 months post‐RP vs. 22% in our study) [8]. One reason might be that the LAPPRO population mainly included patients with ≤ pT2 (94%), while ours featured 33% of patients with ≥ pT3. In LAPPRO, however, postoperative radiotherapy did not significantly influence the risk of stenosis within 24 months after surgery. Therefore, and because all stenoses in this study developed within the first 12 months despite further radiotherapy after this period, the authors concluded that postoperative radiotherapy was no significant risk factor for stenosis formation in their study [8].

Our results furthermore highlight a significant impact of the duration of bladder catheterization after RARP on the development of VUAS/US. A normal duration of catheterization, defined in our study as ≤ 8 days, was associated with a lower risk of complications in uni‐ and multivariable analyses. The significant influence of the catheter length of stay was also evident when it was considered as a continuous variable. This is consistent with published evidence: In a small study with open retropubic RP only 1/25 (4%) patients developed US when the catheter was removed on postoperative day 7, compared to 2/16 (13%) and 6/15 (40%) patients whose catheters were removed on postoperative days 14 and 21, respectively (*p* = 0.048) [22]. However, when our patients were subdivided into those with and without radiotherapy, this effect remained only in the non‐radiotherapy subpopulation. Anastomotic insufficiency is usually treated by prolonged catheterization but, however, may lead to urine extravasation which in turn promotes scarring, possibly causing VUAS/US [21, 23]. Radiotherapy, on the other hand, destroys the microvasculature, which can also lead to scarring and VUAS/US [24]. In patients with radiotherapy, normal vs. prolonged catheterization had no effect on the formation of VUAS/US. However, in this subgroup only 16 patients had a prolonged catheterization which is too few to draw reliable conclusions regarding the influence of catheter length of stay. Nevertheless, our findings suggest that minimizing the duration of catheterization may contribute to better outcomes.

The well‐established PCa risk classification of the National Comprehensive Cancer Network (NCCN) subdivides patients with intermediate risk PCa into those with favorable and unfavorable intermediate risk. One considered criterion is the grade group of 1 (GS 3+3 = 6) or 2 (GS 3+4 = 7a) for favorable intermediate risk, and the grade group of 3 (GS 4‐3 = 7b) for unfavorable intermediate risk [25]. We hence subdivided our population into those with GS ≤ 7a and those with GS ≥ 7b and performed our analyses. We found, however, no significant influence of this dichotomized GS on the risk of VUAS/US formation. Also, in a study with mainly open RP and 17% RARP, GS was not a predictor of VUAS in multivariable logistic regression [21].

Despite significance in univariable assessment, our multivariable analysis revealed that pT‐stage, R‐status, and performance of nerve‐sparing surgery did not significantly influence the risk of VUAS/US after RARP. Separate analyses of the subpopulations with and without radiotherapy support this finding, as even the respective univariable analysis showed no differences with respect to pT‐stage and R‐status. However, when evaluating the impact of radiotherapy within the predefined pT‐stage, R‐status, and nerve‐sparing subgroups, performing radiotherapy proved to be associated with an increased risk of VUAS/US. In a single center retrospective study of 265/294 (90%) PCa patients with pT2 and 29/294 (9.9%) with pT3 who underwent retropubic RP, no significant correlation was found between anastomotic stricture formation and tumor stage. The same applied for R‐status [23]. In the aforementioned study [21], a higher pT stage (pT2 vs. pT3a and pT2 vs. pT3b/4) was a predictor of VUAS within the first year post‐RP, while nerve‐sparing surgery reduced the risk of VUAS. For the latter, a plausible explanation is that nerve‐sparing preserves more perianastomotic tissue, resulting in improved perfusion. This is consistent with our finding that bilateral nerve‐sparing reduces the risk of VUAS/US compared with no nerve‐sparing. The apparently better outcomes without nerve‐sparing than with unilateral nerve‐sparing are likely due to the small number of events and thus unstable estimates. After approximately 5 years, a clear divergence in the Kaplan–Meier curves becomes apparent: patients undergoing bilateral nerve‐sparing exhibit the most favorable long‐term outcomes, followed by those with unilateral nerve‐sparing and those without nerve preservation. This trend is in line with what might be expected. Consistent with our results, the R‐status was not predictive in the context of the referenced trial [21]. However, it should be noted that this study included a mixed population of patients with open and robotic‐assisted RP, with the surgical technique also identified as a predictive factor for VUAS formation. This is an important difference from our RARP‐only population.

One limitation of our study is its retrospective design with potential implications for bias, variability in follow‐up, and lack of control for potential confounding factors. Additionally, the very limited number of events available may lead to overfitting and unstable estimates, particularly in multivariable models. As diagnosis of VUAS/US was symptom‐based, asymptomatic cases may have been missed. The same applies to an unknown number of symptomatic patients who did not undergo urethrocystoscopy and to patients lost to follow‐up. We conclude that early diagnosis of PCa should be sought also, but not exclusively to avoid VUAS/US. If RARP is indicated, this could reduce the need for additional therapies such as adjuvant radiotherapy and thus reduce the risk of VUAS/US in this setting. An uncomplicated procedure may also have a positive effect on the length of catheterization, which has been identified as a risk factor. It is important that RARP patients and clinicians are made aware of the potential symptoms of VUAS/US and that patients are advised to consult their urologist if symptoms occur. This counseling should be repeated if radiotherapy is performed.

## Author Contributions

**Kinan Massouh:** conceptualization, investigation, writing – original draft, methodology, visualization, writing – review and editing, formal analysis, data curation, resources, project administration. **Katharina Leucht:** conceptualization, writing – original draft, methodology, validation, visualization. **Lutz Leistritz:** formal analysis, software, visualization. **Marc‐Oliver Grimm:** conceptualization, software, resources, supervision, data curation, visualization, investigation, methodology, validation.

## Ethics Statement

The protocol for this research project has been approved by a suitably constituted Ethics Committee of the institution, and it conforms to the provisions of the University of Jena. Protocol Nr. 2025‐3739‐BO‐D.

## Consent

Before entering the study, all patients provided written informed consent.

## Conflicts of Interest

Kinan Massou and Katharina Leucht declare no conflicts of interest. Marc‐Oliver Grimm is an Editorial Board member of International Journal of Urology and a co‐author of this article. To minimize bias, they were excluded from all editorial decision‐making related to the acceptance of this article for publication.

## Supporting information


**Table S1:** iju70339‐sup‐0001‐TableS1.docx.
