# Supplementary material for: Retrospective Analysis of Vesicourethral‐Anastomosis Stricture/Urethral Stricture After Robotic‐Assisted Laparoscopic Radical Prostatectomy With and Without Radiotherapy
Source: Int J Urol. 2026 Jan 11;33(1):e70339. doi: 10.1111/iju.70339 (PMC12792238; doi:10.1111/iju.70339)
Supplement: Supplementary file 1 — Table S1: iju70339‐sup‐0001‐TableS1.docx. [file IJU-33-0-s001.docx]

**Supplementary Table 1: Univariable analysis of time to formation of vesicourethral anastomosis strictures (VUAS) and/or urethral strictures (US) in patients with or without radiotherapy and varying risk factors.**

^1)^ Derived via Kaplan-Meier method, after 5 years; CI, confidence interval; NE, not estimable; NS, nerve-sparing; R0, negative tumour margin, R1, positive tumour margin; RT(-), without radiotherapy; RT(+), with radiotherapy.

| **Risk factor** | **Risk factor 1** | **No. of patients** | **Risk factor 2** | **No. of patients** | **VUAS/US, %^1)^** | **95% CI** | **p-value** |
| --- | --- | --- | --- | --- | --- | --- | --- |
| **pT-stage** | RT(-) | 634 | ≤pT2c | 485 | 1.6 | 0.4, 2.8 | 0.119 |
|  |  |  | ≥pT3a | 149 | 3.8 | 0.5, 7.1 |  |
|  | RT(+) | 175 | ≤pT2c | 56 | 7.8 | 0.4, 15.2 | 0.749 |
|  |  |  | ≥pT3a | 119 | 7.0 | 2.3, 11.7 |  |
|  | ≤pT2c | 541 | RT(-) | 485 | 1.6 | 0.4, 2.8 | <0.001 |
|  |  |  | RT(+) | 56 | 7.8 | 0.4, 15.2 |  |
|  | ≥pT3a | 268 | RT(-) | 149 | 3.8 | 0.5, 7.1 | 0.045 |
|  |  |  | RT(+) | 119 | 7.0 | 2.3, 11.7 |  |
| **Gleason Score** | RT (-) | 634 | ≤7a | 434 | 2.3 | 0.7, 3.9 | 0.640 |
|  |  |  | ≥7b | 200 | 1.5 | -0.3, 3.3 |  |
|  | RT (+) | 175 | ≤7a | 52 | 9.1 | 0.5, 17.7 | 0.792 |
|  |  |  | ≥7b | 123 | 6.6 | 2.1, 11.1 |  |
|  | ≤7a | 486 | RT (-) | 434 | 2.3 | 0.7, 3.9 | 0.002 |
|  |  |  | RT (+) | 52 | 9.1 | 0.5, 17.7 |  |
|  | ≥7b | 323 | RT(-) | 200 | 1.5 | -0.3, 3.3 | 0.0004 |
|  |  |  | RT (+) | 123 | 6.6 | 2.1, 11.1 |  |
| **R-status** | RT(-) | 634 | R0 | 529 | 2.1 | 0.7, 3.5 | 0.968 |
|  |  |  | R1 | 105 | 1.9 | -0.6, 4.4 |  |
|  | RT(+) | 175 | R0 | 68 | 4.6 | -0.5, 9.7 | 0.440 |
|  |  |  | R1 | 107 | 9.0 | 3.3, 14.7 |  |
|  | R0 | 597 | RT(-) | 529 | 2.1 | 0.7, 3.5 | 0.006 |
|  |  |  | RT(+) | 68 | 4.6 | -0.5, 9.7 |  |
|  | R1 | 212 | RT(-) | 105 | 1.9 | -0.6, 4.4 | 0.009 |
|  |  |  | RT(+) | 107 | 9.0 | 3.3, 14.7 |  |
| **Nerve-sparing** | RT(-) | 634 | No NS | 94 | 2.7 | -1.0, 6.4 | 0.563 |
|  |  |  | Unilateral | 107 | 3.1 | -0.4, 6.6 |  |
|  |  |  | Bilateral | 433 | 1.7 | 0.5, 2.9 |  |
|  | RT(+) | 175 | No NS | 55 | 7.7 | 0.4, 15.0 | 0.119 |
|  |  |  | Unilateral | 31 | 11,2 | -1.0, 23.4 |  |
|  |  |  | Bilateral | 89 | 5.7 | 0.8, 10.6 |  |
|  | No NS | 149 | RT(-) | 94 | 2.7 | -1.0, 6.4 | 0.005 |
|  |  |  | RT(+) | 55 | 7.7 | 0.4, 15.0 |  |
|  | Unilateral | 138 | RT(-) | 107 | 3.1 | -0.4, 6.6 | 0.120 |
|  |  |  | RT(+) | 31 | 11 | -1.0, 23.4 |  |
|  | Bilateral | 522 | RT(-) | 433 | 1.7 | 0.5, 2.9 | 0.004 |
|  |  |  | RT(+) | 89 | 5.7 | 0.8, 10.6 |  |
| **Duration of bladder catheteri-zation** | RT(-) | 634 | Normal | 586 | 1.5 | 0.5, 2.5 | 0.001 |
|  |  |  | Prolonged | 48 | 8.9 | 0.7, 17.1 |  |
|  | RT(+) | 175 | Normal | 159 | 6.7 | 2.8, 10.6 | 0.624 |
|  |  |  | Prolonged | 16 | 13 | -4.0, 29.8 |  |
|  | Normal | 745 | RT(-) | 586 | 1.5 | 0.5, 2.5 | <0.001 |
|  |  |  | RT(+) | 159 | 6.7 | 2.8, 10.6 |  |
|  | Normal | 64 | RT(-) | 48 | 8.9 | 0.7, 17.1 | 0.594 |
|  |  |  | RT(+) | 16 | 13 | -4.0, 29.8 |  |
